# Cancer Screening in Refugees and Immigrants: A Global Perspective

**DOI:** 10.4269/ajtmh.21-0692

**Published:** 2022-03-09

**Authors:** Patricia F. Walker, Ann M. Settgast, Malini B. DeSilva

**Affiliations:** ^1^ HealthPartners Institute, Bloomington, Minnesota;; ^2^ Department of Medicine, University of Minnesota, Minneapolis, Minnesota;; ^3^ HealthPartners Center for International Health, St. Paul, Minnesota;; ^4^ HealthPartners Travel and Tropical Medicine Center, St. Paul, Minnesota

## Abstract

Clinicians in the United States are trained to screen for cancer based on patient age, gender, family history, and environmental risk factors such as smoking. These cancers generally include, breast, cervical, colon, lung, and prostate cancers. We know that refugees and other immigrants to the United States experience dramatic disparities in cancer screening. Additionally, many immigrants experience elevated risks from infection-attributable cancers due to their country or region of origin. U.S.- based clinicians may not routinely consider these unique risk factors. Although this article focuses on refugees, it is also intended to guide clinicians caring for other foreign-born immigrant groups living in the United States (hereafter referred to as “immigrants”). The document contains two sections: 1) special considerations for U.S. Preventive Services Task Force guidelines cancer screening recommendations in immigrants and 2) cancer risks and screening recommendation unique to certain immigrant groups. Disparities in cancer screening and prevalence are often greater for specific immigrant groups than for broader racial or ethnic groups (e.g., Black, Asian, Hispanic) into which they may fit. Disaggregation of data by language or country of origin is useful to identify such disparities and to design intervention opportunities within specific communities that are culturally distinct and/or who have different environmental exposures. Unique cancer risks and disparities in screening support a nuanced approach to cancer screening for immigrant and refugee populations, which is the focus of this narrative review.

## INTRODUCTION

Many immigrants experience elevated risks from infection-attributable cancers based on their country or region of origin.^1^ Additionally, refugees in the United States experience dramatic disparities in cancer screening.^2^^,^^3^ While refugees are a particularly vulnerable subset of immigrants, we recognize that they comprise a small percentage of immigrants living in the United States. Thus, this document is intended to guide clinicians caring for both refugees and other foreign-born immigrant groups living in the United States (hereafter referred to as “immigrants”). The article contains two sections: 1) special considerations for U.S. Preventive Services Task Force guidelines (USPSTF) cancer screening recommendations in immigrants (Table 1) and 2) cancer risks unique among certain immigrant groups (Table 2).

**Table 1:** Key considerations for cancer screening in immigrants for which there are U.S. Preventive Services Task Force guidelines

Breast cancer	Immigrant women in the United States undergo mammography at lower rates than U.S.-born women despite breast cancer being the leading cause of cancer death in most low- and middle-income countries. Clinicians and health systems should spend extra time and effort to tailor education to immigrant and refugee women to address this inequity.
Colon cancer	Colonoscopy is the least completed cancer screening test among immigrant groups. Colon cancer screening rates vary dramatically between immigrant groups underlying the need for targeted approaches to improve colonoscopy uptake.
Cervical cancer	It is especially important to perform Pap screening on older refugee and immigrant women because those aged over 65 years who have never been screened with Pap smears have the highest mortality from cervical cancer and benefit most from screening. The common practice in the United States of ceasing Pap screening at age 65 years does not apply to the vast majority of refugee and immigrant women because they do not have a history of negative prior screening. Women over 65 years who have never been screened should have 10 years of negative cervical cancer screening before cessation of screening. Current guidelines recommend screening initiation at age 21 years regardless of age of sexual debut (i.e., first intercourse). However, Pap screening before sexual debut in young women with infibulation (i.e., type III female genital cutting [FGC]) may not be anatomically feasible. Screening in women who have undergone FGC and have experienced their sexual debut should not differ from women without FGC history.
Lung cancer	Obtain careful tobacco use history from immigrant patients, recognizing the wide variation and often very high rates of smoking in some groups. Recognize that some refugees and immigrants who have never smoked may have higher risk for lung cancer given high rates of air pollution, exposure to indoor biomass smoke, radon, arsenic and asbestos.

**Table 2:** Key considerations for cancer screening in immigrants for which there are no U.S. Preventive Services Task Force guidelines.

Hepatocellular carcinoma (HCC)	Screen all refugees and immigrants born in countries with greater than 2% hepatitis B virus (HBV) prevalence, if not completed overseas before U.S. arrival. Perform hepatitis C virus screening for all individuals 18–79 years of age, and those with known risk factors. All HBV and HCV infected individuals should be evaluated by a hepatologist and should undergo HCC screening in accordance with national guidelines, which includes initiation of HCC surveillance at age 20 years for African-born patients with chronic HBV. HCC screening includes laboratory testing and ultrasound or other imaging modalities every 6 months. Although not part of the America Association for the Study of Liver Disease guidelines, given increasing HCC rates in Asian immigrants < 30 years, clinicians may consider initiation of HCC screening for Asian patients with chronic HBV infection at age 20 years. Automated best practice alerts that trigger based on country of birth can improve screening for HBV; linkage to a primary care provider, implementation of a chronic disease registry for HBV, and use of culturally tailored educational materials also improves adherence to screening recommendations.
Gastric cancer	Gastric cancer incidence varies dramatically worldwide, and many immigrants come from high-incidence countries. No U.S. guidelines exist regarding screening for gastric cancer in high-risk immigrant populations, despite implementation of successful screening programs in some high-risk countries. Identify patients at high-risk for gastric cancer based on ethnicity, country of origin, family history of gastric cancer, or *Helicobacter pylori* infection. Consider screening patients at high risk for gastric cancer with endoscopy, and treat symptomatic, infected patients to eradicate *H. pylori*.
Bladder cancer	No screening recommendations exist for patients with *Schistosoma hematobium*, a known risk factor for bladder cancer. Immigrant patients from endemic areas who present with urinary symptoms (e.g., dysuria, gross hematuria, pelvic pain) should be screened for hematuria with urinalysis, and, if present, evaluate further with urine cytology, urine ova, and parasite testing (between 12 and 3 pm), serology for schistosomiasis and cystoscopy. Patients from S. hematobium–endemic areas with unexplained hematuria should be referred for cystoscopy and considered for empiric treatment with praziquantel due to potential benefits vs. risk of treatment, and low sensitivity of testing.
Cholangiocarcinoma	Identify high risk groups for biliary tract cancers due to liver fluke infection based on region of origin (Southeast Asia, including northern Thailand, northern Vietnam and Laos, Manchuria, east Russia and northern Siberia, South Korea, mainland China except the northwest, and Taiwan), and exposure history (eating raw or fermented freshwater fish). Evaluate for liver fluke infection with complete blood count with differential and three stools for ova and parasite testing in patients from endemic areas with a history of biliary tract stones or dilated intrahepatic bile ducts without obstruction. Consider empiric treatment with praziquantel for patients from endemic areas with a history of biliary stones or dilated intrahepatic bile ducts due to potential benefits vs. risk of treatment, and low sensitivity of stool testing for ova and parasites.
Nasopharyngeal cancer	Consider screening high-risk persons with serology, clinical examination, and nasopharyngoscopy—those from southern China (including Hong Kong), Singapore, Malaysia, Philippines, and Vietnam, non-U.S. born Hmong individuals, and those with a family history of nasopharyngeal cancer. Patients at high-risk for nasopharyngeal cancer presenting with persistent nasal obstructive symptoms, discharge, epistaxis, tinnitus, or hearing loss should undergo careful physical examination for adenopathy and early referral to an otorhinolaryngology specialist rather than empiric treatment of symptoms.
Oral and esophageal cancer	Screen for use of betel nut and areca nut in addition to tobacco products and perform a thorough oral examination on an annual basis. Early referral to otorhinolaryngology for evaluation of suspicious findings, including leukoplakia, erythroplakia, or oral submucous fibrosis. Counsel on cessation of use of betel nut and areca quid, as well as other tobacco products.

Disparities in cancer screening and prevalence are often greater for specific immigrant groups, than for broader racial or ethnic groups (e.g., Black, Asian, Hispanic) into which they may fit. Disaggregation of data to country-of-origin level is useful to identify such disparities and design intervention opportunities within specific communities that are culturally and genetically distinct with different environmental exposure histories.^4^ Unique cancer risks and disparities in screening support a nuanced approach to cancer screening for immigrant populations, which is the focus of this narrative review.

Lack of symptoms contributes to underscreening in many patient populations, particularly in immigrant populations who have not had regular access to routine cancer screening.^5^ A recent systematic review of barriers to breast and cervical cancer screening among U.S. immigrants found that lack of both health insurance and a usual source of care were the most prominent barriers to cancer screening.^6^ Lack of knowledge of screening procedures is another common barrier.^5^ Community-based programs using culturally sensitive approaches tailored to individual ethnic groups within broader immigrant communities have proven successful in improving cancer screening behavior.^7^

## SPECIAL CONSIDERATIONS FOR USPSTF CANCER SCREENING GUIDELINES IN REFUGEES AND IMMIGRANTS

### Breast cancer screening.

Breast cancer is the most common cancer among women worldwide with incidence increasing in all regions. It is the leading cause of cancer death among women in many low- and middle-income countries (LMIC), despite lower incidence in these regions.^8^ There is conflicting information regarding rates of breast cancer in immigrant groups compared with U.S.-born populations with some studies showing lower rates^9^ and others showing higher.^10^ For many cancers, risk for immigrant patients increases with increasing time in an industrialized setting (i.e., reversal of the “healthy migrant effect”).^11^

Although there are sizeable disparities in mammogram uptake in immigrant groups compared with U.S.-born women, increased time in the United States correlates positively with mammogram uptake. Long-term U.S. residence can even be associated with higher screening rates than in U.S.-born women.^12^ Although not specific to immigrants, racial and ethnic minority women in the United States present with later-stage breast cancer and have higher mortality rates than non-Hispanic Whites.^13^ Special efforts to improve breast cancer screening for all immigrant women is needed given their vulnerability to underscreening and the substantial role of breast cancer in women’s health worldwide.

### Colorectal cancer screening.

The USPSTF currently recommends colon cancer screening for individuals aged 45 to 75 years.^14^ Some immigrant groups in the United States may have lower risk of colorectal cancer than nonimmigrant populations.^15^ However, as with breast cancer, it is worth noting that immigrants in the United States have significantly lower rates of colorectal cancer *screening* compared with U.S.-born adults regardless of time since immigration. As with breast cancer screening, longer duration of residence has been shown to correlate with improved screening. Only 36.3% of age-eligible immigrants residing in the United States less than 10 years reported colorectal cancer screening versus 52.3% residing in the United States for more than 10 years.^16^ Late presentation and poor outcomes are also features of colon cancer in racial and ethnic minority populations in the United States.

Although there are parallels between breast and colon cancer screening data in immigrants, colon cancer poses special challenges. One study revealed that colonoscopy is the least performed screening test among immigrant women.^17^ In a study describing a targeted intervention to improve cancer screening in distinct refugee groups, colon cancer screening had the lowest uptake compared with screening for breast and cervical cancer.^7^ In Minnesota, colon cancer screening among adults overall is 71%, but only 40% of Hmong speakers and 32% of Somali speakers completed screening.^18^ Increased time residing in the United States may partially explain high rates of screening (73%) among Vietnamese speakers in Minnesota.^18^ Such impressive disparities found in the disaggregated data support the need for targeted approaches to cancer screening for distinct immigrant groups.

### Cervical cancer screening.

Cervical cancer is the leading cause of cancer death in women in dozens of countries, most of which are in sub-Saharan Africa.^19^ This is related to differing access to Pap screening (only 5% of women in LMICs have undergone cervical cancer screening in the past 5 years)^20^ and human papilloma virus (HPV) vaccination.^21^ Although HPV vaccine has dramatic benefits in reducing early cervical disease, its use lags in regions of the world where it would have the greatest impact. By early 2020, < 30% of LMICs had implemented national HPV vaccination programs compared with > 80% of high-income countries.^19^

Immigrant women are more than twice as likely as U.S.-born women to have never received Pap screening (18.6 versus 6.8%) even after controlling for socioeconomic differences and differences in healthcare access and utilization.^3^ This renders them particularly high risk for cervical cancer. As with breast and colon cancer, longer amounts of time residing in the United States increases screening uptake, with those residing in the United States more than 10 years having greater odds of having had Pap testing than those residing in the United States for less than 10 years.^22^

Women over age 65 who have never undergone cervical cancer screening have the highest mortality from cervical cancer and benefit most from screening.^23^^,^^24^ The USPSTF recommends cervical cancer screening cease at age 65 in women who have undergone adequate prior screening with negative results. However, this does not apply to most immigrant females because the number of immigrant women older than 65 who have never been screened is nearly triple that of U.S.-born women in the same age category (17.1% versus 6%).^3^ Cervical cancer screening of immigrant women over age 65 is strongly indicated unless they have documented adequate screening for one decade prior to cessation.

In the United States, initiation of Pap screening is recommended at age 21 regardless of sexual history. However, women who have undergone type III female genital cutting (FGC; i.e., infibulation) require special consideration. Rates of infibulation vary greatly among immigrant women with some groups (e.g., Somali women) experiencing prevalence of 98%.^25^ Infibulation does not protect against cervical cancer, and sexual activity in this group of women confers risk of HPV infection and cervical cancer as it does in women without infibulation. In one study of East African women, the prevalence of severe cervical dysplasia (high-grade squamous intraepithelial lesion positive) was 2.6%, irrespective of FGC history.^26^ Thus, in women with infibulation who have been sexually active, Pap testing should be performed according to the usual USPSTF schedule.

In women with infibulation seen before their sexual debut, decisions related to attempting HPV testing alone should be made on an individual basis within a clinician-provider relationship grounded in excellent communication. For example, in a 30-year-old woman with infibulation and a history of nonpenetrative sexual activity (but before her sexual debut), attempts at testing may be appropriate given her potential risk of HPV infection and thus cervical cancer. On the other hand, in a 30-year-old woman with infibulation who has never experienced any form of sexual activity, Pap screening is generally anatomically infeasible, fails to provide any health benefit, may be traumatic, and thus is not recommended.

### Lung cancer screening.

Lung cancer is the largest contributor to cancer mortality in the world.^27^ The USPSTF recommends annual screening with low-dose computed tomography (CT) in adults aged 50 to 80 years who have a 20 pack-year history and currently smoke or who have quit within the past 15 years. Smoking rates among immigrants vary with some groups having higher and others lower smoking rates compared with the general U.S. population.^28^ However, it is worth noting that tobacco use patterns are evolving globally: by 2016, 80% of smokers aged ≥ 15 years resided in LMICs.^19^ Given these data, certain immigrant groups may be at particularly high risk of lung cancer. Therefore, as with all patients, regardless of country of origin, accurate smoking history should be obtained from immigrants to make appropriate screening recommendations with consideration of annual low dose CT scan.

In addition, an estimated 10% to 15% of lung cancers occur in patients who have never smoked. Certain immigrants may be at higher risk for lung cancer given environmental exposure to radon, arsenic, asbestos, indoor biomass smoke or high levels of air pollution.^29^

## CANCERS WITH HIGHER PREVALENCE IN CERTAIN REFUGEE AND IMMIGRANT POPULATIONS

The majority of unique cancer risk in refugee and immigrant groups originates from cancers attributable to infection. Of the 2.2 million new cancer cases worldwide in 2018, 13% were attributable to infection.^1^ However, variation by region is dramatic, with only 4% of cancers in North America being infection-related compared with 31.3% in sub-Saharan Africa. *Helicobacter pylori*, HPV, hepatitis B virus (HBV), and hepatitis C virus (HCV) account for 92% of all infection-attributable cancers worldwide.^6^^,^^21^ Country-specific cancer data are available from the Global Cancer Observatory at the WHO.^30^ A study of cancer mortality among U.S.-born and immigrants in the United States during 2005–2014 found immigrants had higher cancer mortality rates for seven cancer sites, five of which were infection related (nasopharyngeal, Kaposi’s sarcoma, stomach, liver, and intrahepatic bile duct).^31^

### Hepatocellular cancer (HBV, HCV, and schistosomiasis).

In 2018, 841,000 people worldwide were diagnosed with hepatocellular carcinoma (HCC).^8^ Liver cancer causes more than 782,000 deaths annually worldwide.^8^ The incidence of liver cancer is rising globally, primarily because of limited progress in viral hepatitis prevention.^32^ During 2005–2014, liver cancer mortality rates among immigrants in the United States were consistently higher than U.S.-born populations.^33^ Independent risk factors for development of HCC include increasing age, male gender, elevated HBV DNA levels, history of reversion to hepatitis B e antigen positivity, HBV genotype C, coinfection with HCV, core promoter mutations, and presence of cirrhosis.^34^ The estimated risk for development of HCC in patients with HBV or HCV-associated chronic active hepatitis, cirrhosis or both is as high as 25%.^35^^,^^36^ The long-term prognosis for patients diagnosed with HCC remains poor, with a 5-year survival rate of 10% to 12% in the United States and even lower survival rates in LMICs, where more than 80% of HCC cases occur.^32^^,^^37^ Whereas the incidence of HCC in the United States has primarily risen due to HCV,^34^ immigrants in the United States are at higher risk for HCC due to higher rates of HBV infection.^35^ In addition, some ethnic groups have higher rates of liver cancer related to infection with HCV, *Schistosoma mansoni*, or *Schistosoma japonicum*.^38^^–^^40^

### Hepatitis B.

Both the USPSTF and the U.S. Centers for Disease Control and Prevention recommend screening populations at increased risk for HBV infection including those born in countries with ≥ 2% HBV prevalence; nearly all U.S.-bound refugees arrive from such countries. An estimated 3.5 million refugees worldwide have chronic HBV infection.^41^ Asian Americans have the highest incidence of HCC of all ethnic groups in the United States, and as many as 10% of non U.S.-born Asian Americans are chronically infected with HBV.^42^ Among people with untreated chronic HBV infection, 15% to 40% will develop cirrhosis, hepatic decompensation, or HCC.^35^^,^^36^ In addition, patients who spontaneously clear hepatitis B surface antigen (HBsAg) may develop HCC. In one study, 6.5% of patients followed for a median of 56 months after clearing HBsAg developed HCC.^43^

### Screening for HCC in HBV-infected patients.

The American Association for the Study of Liver Diseases practice guidelines recommend screening patients with chronic HBV for liver cancer with ultrasound every 6 months for selected patient populations, with onset varying by region of origin and age.^44^

HCC screening guidelines vary regarding use of alpha-fetoprotein (AFP) as a screening adjunct.^45^ A meta-analysis of studies comparing the performance of ultrasound alone versus ultrasound plus AFP for early HCC detection found concomitant use of ultrasound and AFP improved early HCC detection compared with ultrasound alone, with sensitivities of 63% (95% confidence interval [CI]: 48–75%) and 45% (95% CI: 30–62%), respectively.^46^ Using an AFP trend value rather than a single test result can also more accurately identify patients with early stage HCC.^47^^,^^48^ Consistent increases in AFP level, even if below normal values of < 20 ng/mL, may be concerning and should prompt further imaging studies.^49^

Early-onset HCC (diagnosis at age < 30 years), related to HBV infection is more common in African-born patients^50^; this may be due in part to age of acquisition of infection, higher exposure to aflatoxins or to HBV genotype.^51^ The U.S. guidelines now recommend initiation of HCC surveillance at age 20 for African born patients with chronic HBV.^51^ In younger Asian immigrants, family history and smoking history, even in the absence of cirrhosis, may identify those at higher risk for development of HCC. In addition, subtype B2 accounts for 15% to 20% of all HCC cases in Asia,^52^ and its incidence is increasing.^53^ Due to increasing rates of early-onset HCC among persons of Asian descent, clinicians may consider initiating screening for Asian patients at age 20, earlier than current guidelines recommend.^52^^,^^54^

### Adherence to practice guidelines for liver cancer screening in patients with HBV.

Adherence to clinical guidelines for HCC screening in patients with HBV infection is suboptimal among healthcare providers, often below 50%.^55^^,^^56^ Higher screening rates were reported among providers caring for more Asian patients and who have increased knowledge regarding refugee health care.^55^ Improved HCC screening rates and linkage to care for long-term management of HBV infection has been achieved through use of culturally tailored educational interventions (e.g., translated materials and ethnically concordant community health worker outreach) and increased linkage to primary care.^57^^,^^58^ Additionally, automated best practice alerts that trigger based on country of birth can improve screening for HBV, and implementation of a chronic disease registry for HBV may improve adherence to HBV care and HCC screening recommendations.^59^

### Hepatitis C.

Although hepatitis C is a risk factor for hepatocellular carcinoma, there is little generalizable data on the epidemiology of HCV infection in refugee populations. Prevalence rates vary among refugee groups from very low (< 1%) to high (7–8%), and further research is needed to define HCV infection rates in refugees. Two refugee groups of particular interest are Burmese and Hmong born in Thailand, with HCV prevalence of approximately 7%, and Somali refugees, who have a high prevalence of HCC related to HBV and HCV.^60^ In 2020, the CDC recommended HCV screening for all individuals 18 years and older at least once in a lifetime.^61^ In the United States, patients diagnosed with HCV and cirrhosis should undergo routine surveillance for HCC.^51^

### Schistosomiasis.

*Schistosoma mansoni* leads to liver fibrosis, and may be linked to HCC both as an independent risk factor, and through potentiating the effects of HCV or HBV on the liver.^38^^–^^40^ *Schistosoma japonicum* is also a risk factor for liver and colorectal cancer.^37^ Although no specific guidelines exist for screening for liver cancer in patients infected with *S. mansoni* and *S. japonicum*, clinicians should be aware of the association between infection with certain *Schistosoma* spp. and liver and colon cancer.

### Gastric cancer (*Helicobacter pylori*).

In 2018, gastric cancer was the sixth most common cancer and the second most common cause of cancer-related deaths worldwide.^62^ Incidence rates vary dramatically by region, with East Asia having the highest rate. There are currently no clear U.S. guidelines regarding screening for gastric cancer in immigrants from high-risk countries.

However, gastric cancer screening has been implemented in several countries with high incidence rates, including Japan, South Korea, and China.^63^ A meta-analysis of outcomes from these countries found that screening programs were associated with a 40% reduction in gastric cancer mortality and were cost effective.^63^

Risk factors for gastric cancer include *Helicobacter pylori* infection, host genetic factors, and environmental factors such as high intake of salty and pickled foods. *H. pylori* is a Class I carcinogen and is responsible for 60% to 80% of all gastric cancers of intestinal and diffuse type, as well as gastric mucosa-associated lymphoid tissue lymphoma.^64^ Randomized controlled trials have provided evidence for the effectiveness of *H. pylori* identification and eradication in preventing gastric cancer.^65^ Despite the known association between *H. pylori* and gastric cancer, routine screening for *H. pylori* is not recommended.

The American Gastroenterological Association recommends against routine use of endoscopic surveillance in patients with gastric intestinal metaplasia, instead advocating surveillance only in high-risk groups, including individuals with a family history of cancer, extensive mucosal involvement (spanning both the gastric body and antrum rather than being limited to the antrum alone), and racial/ethnic minorities or immigrants from high-risk regions.^66^ One review article recommended considering screening endoscopy for individuals with known risk factors for gastric cancer including immigrants from East Asia, Russia, and South America, or who have a family history of gastric cancer.^67^ A 2020 commentary outlined a theoretical approach whereby screening endoscopy could be considered for patients coming from countries where reported incidence and mortality for esophageal and gastric cancer were within 20% of U.S. rates for colon cancer incidence and mortality (Table 3).^63^

**Table 3:** Countries with age-standardized incidence of gastric or esophageal cancer greater than or within 20% of U.S. colorectal cancer (CRC) rates stratified by biologic sex.

	Male	Female
U.S. CRC incidence	23.1 per 100,000 population	17.1 per 100,000 population
Countries with age-adjusted esophageal and gastric cancer incidence greater than U.S. CRC rates (rate per 100,000 population)	Mongolia (56.3) Republic of Korea (54.5) China (40.9) Japan (38.4) Republic of Cabo Verde (33.7) Kazakhstan (28.9) Bhutan (27.7) Tajikistan (26.6) Kenya (26.2) Democratic People’s Republic of Korea (25.8) Myanmar (25.4) Lithuania (24.2) Vietnam (23.6) Turkmenistan (23.5) Latvia (23.5)	Mongolia (28.8) Kenya (21.4) Republic of Korea (20.7) Bhutan (17.3) Malawi (17.1)
Countries with age-adjusted esophageal and gastric cancer incidence within 20% of U.S. CRC rates (rate per 100,000 population)	Bangladesh (23.1) Russian Federation (23) Malawi (22.3) Chile (21.9) Lao People’s Democratic Republic (21.8) Ukraine (21.6) Azerbaijan (21) Estonia (20.9) Iran (20.7) Moldova (20.6) Reunion (19.5) Slovakia (19.2) Portugal (19) Hungary (18.5)	Tajikistan (17.1) China (16.5) Zimbabwe (16.5) Japan (13.8)

Adapted from Laszkowska et al.^63^

Ultimately, there are no clear screening guidelines for clinicians caring for immigrants from high-risk regions; clinicians should be aware of the elevated risk for gastric cancer, and work collaboratively with GI colleagues on evaluation of high-risk patients.

### Bladder cancer (schistosomiasis).

In 2018, there were an estimated 549,000 new cases of bladder cancer worldwide; the majority occurred in males.^62^ Smoking and occupational exposure are the major risk factors in higher income countries, whereas in LMICs, particularly the Middle East and Africa, chronic infection with *Schistosoma hematobium* is a primary risk factor. The USPSTF notes current evidence is insufficient to assess the balance of benefits and harms of bladder cancer screening.^68^ Nevertheless, clinicians seeing patients from *S. hematobium*–endemic countries with urinary symptoms, particularly hematuria, may consider further evaluation with urine cytology, urine ova and parasite testing (between 10 am and 2 pm), serology for schistosomiasis, and cystoscopy to screen for bladder cancer. Given the low sensitivity of laboratory studies for identifying *S. hematobium* and the potential benefit versus risk of treatment, some clinicians empirically treat patients from endemic areas with unexplained hematuria with praziquantel.

### Cholangiocarcinoma (liver flukes).

*Clonorchis sinensis*,* Opisthorcis viverrini*, and *Opisthorcis felineus* are trematodes (flukes) that infect the biliary tract and are transmitted through ingestion of raw or partially cooked freshwater fish infected with metacercariae. Approximately 35 million people are infected worldwide. Endemic areas for *C. sinensis* include Korea, China, Taiwan, northern Vietnam, and far eastern Russia. Endemic areas of *O. viverrini* include Laos and northeast Thailand, whereas *O. felineus* is endemic in Eastern Europe and the former USSR.^63^ Adult flukes may live for 20 to 30 years in the intrahepatic bile ducts and may also live in the common bile duct, gallbladder, or the peripheral pancreatic ducts. Unless heavily infected, patients are asymptomatic. Flukes cause chronic inflammation of the bile ducts, leading to suppurative cholangitis, bile duct stones, and cholangiocarcinoma. Cholangiocarcinoma is relatively uncommon in the Western Hemisphere with incidence rates of 0.2 to 0.7 per 100,000 people.^69^ In certain parts of Asia, such as northern Thailand and Korea, the incidence is much higher, 84.6 per 100,000 and 7.4 per 100,000, respectively, which is related to the high prevalence of opisthorchiasis and clonorchiasis.

The characteristic radiological finding of previous liver fluke infection is diffuse dilatation of the peripheral intrahepatic bile ducts without obstruction on imaging.^70^ Patients from endemic areas with unexplained intrahepatic bile duct dilatation on imaging should undergo screening for eosinophilia and stool ova and parasites. Due to low sensitivity of these tests, and the potential benefit versus risk, some clinicians empirically treat patients from endemic areas with a history of biliary stones or unexplained dilated intrahepatic bile ducts with praziquantel.

### Nasopharyngeal cancer (Epstein-Barr virus).

Nasopharyngeal cancer (NPC) has striking epidemiologic features, including regional, ethnic, and familial aggregations.^71^ NPC occurs more frequently in southern China, Singapore, Malaysia, the Philippines, northwest Canada, and Greenland.^72^ Risk increases slowly throughout life, but NPC can occur at any age. Approximately half of patients with NPC in the United States are younger than 55 years. Men have twice the incidence of women.^73^

The link between NPC and Epstein-Barr virus (EBV) infection is complex. Almost all NPC cells contain EBV, and most people with NPC have evidence of EBV infection. However, EBV infection alone is not enough to cause NPC because infection is common, and NPC is rare. Genetic factors may affect how EBV contributes to the development of NPC.^73^ Other risk factors for NPC include diets high in salt-cured fish and meats, smoking, genetic factors, and family history of NPC. Heavy alcohol use and exposure to formaldehyde or wood dust may also increase the risk of NPC, but data are lacking.^73^

Clinical presentation includes nasal symptoms such as epistaxis, obstruction, or discharge (78%); ear symptoms including infection, deafness, or tinnitus (73%); headaches (61%); regional lymphadenopathy (63%); and cranial nerve palsies.^74^ The most common exam findings are painless bilateral anterior cervical adenopathy (80%), cranial nerve palsies (25%), and nasopharyngeal mass on nasopharyngoscopy.^75^

Screening for NPC remains controversial. A 2011 study of adults (*N* = 42,048) in Guangdong, China, followed patients for 16 years and concluded that early detection of NPC can be achieved by serial serology and clinical examination.^76^ Additionally, some groups recommend screening for NPC among family members who have a relative with NPC using serology, physical examination, and nasopharyngoscopy.^77^ A 2015 Cochrane Collaborative review of 31 randomized controlled trials and controlled clinical trials was unable to determine the efficacy of screening for NPC or the cost-effectiveness of screening.^78^

### Oral and esophageal cancer (HPV and betel quid).

HPV is estimated to cause 70% of oropharyngeal cancers in the United States^79^; one study noted an increased incidence of HPV related cancers in developed countries and at a younger age.^80^ In select immigrant populations, additional oral and esophageal cancer risk factors include betel nut and areca quid, classified as carcinogenic by the WHO. Approximately 10% of the world’s population uses betel nut, including 20% to 40% of the population of India, Nepal, and Pakistan.^72^ Many studies have shown a convincing link between betel nut use and cancer of the mouth and esophagus, including oral squamous cell cancer, leukoplakia, erythroplakia, and oral submucous fibrosis. Among immigrants familiar with betel nut, a majority are aware of the link to cancer, and understanding of risk is improved through use of visually guided educational brochures, suggesting opportunities for interventions.^81^ Cessation counseling is indicated at regular intervals. In addition, clinicians may consider annual visual screening of the oral cavity in tobacco and betel/quid users.

Visual screening of the oral cavity can identify squamous cell carcinoma (SCC) and can improve disease-specific survival, but studies with long-term follow-up are limited.^82^ In a randomized trial of approximately 200,000 patients in India, at up to 9 years of follow-up, visual screening of the oral cavity reduced oral SCC mortality by 27% in all patients and by 29% in ever-tobacco and/or ever-alcohol users.^83^

## CONCLUSION

Early detection of cancer immigrants is an essential component of increasing health equity in the United States. Tables 1 and 2 summarize key issues and approaches to consider in reference to cancer screening in U.S. immigrants.

A culturally and clinically tailored approach to screening for breast, cervical, and colon cancer is recommended in light of the significant deficiencies in screening available before U.S. arrival for most immigrants, coupled with dramatic disparities that occur after immigration. In addition, clinicians should maintain a heightened awareness of cancers related to infectious diseases and environmental exposures that disproportionally impact immigrants and adjust their clinical practices accordingly.
